# Human T-Lymphotropic Virus Type 1 and Cryptococcosis Infection, an Underdiagnosed Association: Case Series and Literature Review

**DOI:** 10.1093/ofid/ofae022

**Published:** 2024-01-12

**Authors:** Fátima Concha-Velasco, Carlos Seas, Eduardo Gotuzzo, Beatriz Bustamante

**Affiliations:** Departamento de Investigación, Universidad Continental, Cusco, Peru; Instituto de Medicina Tropical “Alexander von Humboldt,” Universidad Peruana Cayetano Heredia, Lima, Peru; Departamento de Enfermedades Infecciosas y Tropicales, Hospital Cayetano Heredia, Lima, Peru; Instituto de Medicina Tropical “Alexander von Humboldt,” Universidad Peruana Cayetano Heredia, Lima, Peru; Instituto de Medicina Tropical “Alexander von Humboldt,” Universidad Peruana Cayetano Heredia, Lima, Peru

**Keywords:** Co-infection, Cryptococcal disease, *Cryptococcus*, HIV uninfected, HTLV

## Abstract

Clinical and epidemiological features of 7 human immunodeficiency virus–negative Peruvian patients coinfected with human T-lymphotropic virus type 1 (HTLV-1) and cryptococcosis (2006–2017) were studied. Most cases had meningeal involvement, were male, and originated from Peru's jungle. Patients with cryptococcosis should be tested for HTLV-1 in endemic areas of this retrovirus.

Human T-lymphotropic virus (HTLV) is a retrovirus that affects around 5–10 million people [1–3]. The transmission of HTLV occurs through contaminated blood products in 15%–60% of cases, involving the transfer of HTLV type 1 (HTLV-1)–infected lymphocytes between recipients of whole blood or blood components. Additionally, breastfeeding is responsible for transmission in 10%–20% of cases [4]. Among studied virus subtypes, HTLV-1 and HTLV type 2 (HTLV-2) are better studied, with HTLV-1 associated with several diseases, including inflammatory disease such as HTLV-1–associated myelopathy/tropical spastic paraparesis, hematological malignancy such as adult T-cell leukemia/lymphoma, autoimmune diseases (Sjögren syndrome, uveitis, thyroiditis, arthritis, and infective dermatitis), and opportunistic infections (strongyloidiasis, tuberculosis, and scabies) [5, 6], whereas HTLV-2 does not have any known associated complications [7]. A study in Brazil reported that 0.52%–1.64% of patients with paracoccidioidomycosis and aspergillosis, respectively, were coinfected with HTLV-1 [8]. The immune response to avoid opportunistic infections in people with HTLV-1 infection is hampered by regulatory T cells, which are part of CD4 cells [9].

Cryptococcosis causes 250 000 deaths per year and primarily affects people with HIV (PWH). In people without HIV, the disease predominantly affects the central nervous system and the lungs. Mortality attributable to cryptococcosis is higher in HIV-negative people than in PWH [10].

Currently, the predominant focus of research and documentation on disseminated cases of HTLV-1 infection is from Asian countries, particularly in patients presenting with meningeal, pulmonary, and disseminated cryptococcosis, as evidenced by reports primarily originating from Japan [11–22]. However, in Latin America, despite numerous countries endemic for HTLV-1, clinicians infrequently describe this probable association [23–26].

Peru is an endemic country for HTLV-1, reporting the higher incidence of infection in ethnic groups from Andean (Quechua, Aymara) [27, 28] and from jungle (Shipibo-Conibo, Quechua-Lamas, Wampis, and Amarakaeri) regions [29]. In a case-control study, 85.7% of Peruvians subjects with *Strongyloides stercoralis* hyperinfection had HTLV-1 infection [30]. In addition, León et al reported 4 patients with HTLV-1 and severe Th2 immunosuppression presenting with disseminated paracoccidioidomycosis who acquired the fungal infection in the Peruvian jungle [31, 32]. However, although HTLV-1 is endemic in Peru, until now no description of HTLV-1 infection in subjects with cryptococcosis has been reported. Therefore, the aim of this study was to describe the clinical features of patients with cryptococcosis who tested positive for HTLV-1 in an endemic country for the latter. A literature review of this association was also performed.

## METHODS

This study is a case series conducted at the Alexander von Humboldt Tropical Medicine Institute and Cayetano Heredia Hospital, in Lima, Peru, between January 2003 and December 2017, which serves as a referral hospital for HTLV-1 infection. We enrolled all non-HIV-infected patients with a diagnosis of cryptococcosis, defined as isolation of *Cryptococcus* spp in culture from a sterile site. Medical records of subjects with cryptococcosis were reviewed, and data from patients with an HTLV-1 result were analyzed. The fungal identification was performed by conventional methods (India ink stain, urea assimilation, growth on selective agar *Guizotia abyssinica* at 37°C) at the Tropical Institute Alexander von Humboldt. Cultures were considered negative if *Cryptococcus* spp were not detected at the fifth week of incubation. The L-canavanine-glycine-bromothymol blue medium was used to differentiate *Cryptococcus gattii* from *Cryptococcus neoformans*. HTLV-1 diagnosis was performed using a chemiluminescence test (6L61 Architect HTLV-I/II Reagent Kit, Abbott Diagnostics). The positive samples were confirmed by Western blot analysis.

We attempted to identify all studies regardless of language or publication status (published, unpublished, in press, and in progress) that had reported on the coinfection by these 2 pathogens. The following terms were used for the search: *Cryptococcus*, HTLV-1, and cryptococcal infections in non-HIV-infected populations. The relevant articles from January 1980 to March 2023 were searched at PubMed, Medline, and Embase databases.

The descriptive statistical analysis of clinical, demographic, and laboratory data were made using Stata 17 software (StataCorp, College Station, Texas).

The study protocol was evaluated and approved by the Institutional Research Ethics Committee of the Continental University (approval document number 0149-2023-CIEI-UC).

## RESULTS

During the study period, 339 positive *Cryptococcus* spp culture samples were found in HIV-negative participants and PWH; 38 of them occurred in HIV-uninfected individuals. HTLV-1 infection was identified in 7 subjects with cryptococcal disease. Unfortunately, we did not know how many additional patients with HTLV-1 were tested. However, 7 of 38 cases represent a high percentage (18.4%) of coinfection between HTLV-1 and cryptococcosis, despite not having conducted additional testing of the cases.

The clinical and epidemiological characteristics of the Peruvian subjects and of those identified in the literature are shown in Table 1 [11–23, 25, 26, 28, 33–37].

**Table 1.** Clinical Features, Treatment, and Outcome of Human Immunodeficiency Virus–Uninfected and Nontransplant Subjects With Human T-Lymphotropic Virus Type 1 and Cryptococcal Disease

Reference	No. of Subjects	Sex/Age, y	Origin	Comorbidities	Localization	Clinical Characteristics and Outcome	Laboratorial Characteristics and Other Exams	Antifungal Treatment
Actual report	7	M/61	Arequipa, Peru	None	Meningeal	8-mo history of headache, GCS score 14, stiff neck	CSF: 38 cm H_2_O OP; 11 WBC/μL; glucose 2 mg/dL; proteins 63 mg/dL; India ink: positive; CSF AgCL: 1:512; *Cryptococcus neoformans* isolated from CSF	AmB deoxycholate + FCZ
		F/53	NA	Miliary tuberculosis	Disseminated	NA She died 24 d after hospitalization	*C neoformans* isolated from blood	AmB deoxycholate + FCZ
		M/67	Puno, Peru	Diabetes, HBP, corticoid use	Meningeal	3-mo history of fever, headache, nausea, vomiting. GCS score 11, stiff neck He died 40 d after hospitalization with *Acinetobacter* sp bacteremia	CSF: 15 cm H_2_O OP; 410 WBC/μL; glucose 16 mg/dL; proteins 300 mg/dL; India ink: negative; CSF AgCL: 1:256; *C neoformans* isolated from CSF, with 4.84 CFU/mL log_10_; TFG: 4 d; ADA:1 U/L	AmB deoxycholate + FCZ
		F/43	Huanuco, Peru	Mycosis fungoides, T-cell lymphoma, Scabies	Meningeal	2-wk history of headache, nausea, vomiting, neck stiffness, and weight loss Cured	CSF: 45 cm H_2_O OP; 268 WBC/μL; glucose 39 mg/dL; proteins 252 mg/dL; India ink: negative; CSF AgCL: 1:8; *C neoformans* isolated from CSF; TFG: 7 d	AmB deoxycholate + FCZ
		M/54	Cajamarca, Peru	Disseminated *Strongyloides stercoralis*, *Ancylostoma* spp	Disseminated (pulmonary, gastrointestinal, meningeal)	5-y history of diarrhea, fever, cough, weight loss; 1-wk history of headache Died 21 d after hospitalization with *Acinetobacter* spp bacteremia	CSF: 31 cm H_2_O OP; 2 WBC/μL; glucose 12 mg/dL; proteins 20 mg/dL; India ink: positive; CSF AgCL: 1:4096; *C neoformans* isolated from CSF with 4.72 CFU/mL log_10_ and TFG: 3 d; *C neoformans* isolated from sputum and stool	AmB deoxycholate + FCZ
		F/58	Loreto, Peru	Diabetes, HBP	Meningeal	7-mo history of headache, tremor, weight loss, urinary incontinence Hyperreflexia and meningeal signs Cured	CSF: 27 cm H_2_O OP; 19 WBC/μL; glucose 29 mg/dL; proteins 139 mg/dL; India ink: negative; CSF AgCL: 1:1024; *C neoformans* isolated from CSF; TFG: 8 d; ADA: 14 U/L; chest X-ray: bilateral alveolar infiltrates	AmB deoxycholate + FCZ
		M/47	Lima, Peru	None	Meningeal	12-d history of fever, headache, vomiting, blurred vision Hyperreflexia and meningeal signs Cured	CSF: 42 cm H_2_O OP; 149 WBC/μL; glucose 5 mg/dL; proteins 14 mg/dL; India ink: positive; AgCL: 1:8192; *C neoformans* isolated from CSF with 4.94 CFU/mL log_10;_ TFG: 2 d; ADA: 6 U/L	AmB deoxycholate + FCZ
Altamirano-Molina et al [23]	2	F/28	Lima, Peru	T-cell lymphoma	Lymphadenitis	3-mo history of lymphadenitis, weight loss, hepatosplenomegaly Died	*C neoformans* isolated from blood	AmB deoxycholate + FCZ
		F/68	Lima, Peru	HBP	Lymphadenitis	1-mo history of weight loss Died because of respiratory distress	*C neoformans* isolated from BALF	AmB deoxycholate + FCZ
Motoa et al [24]	1	F/70	Haiti	Coronary artery disease, diabetes, and ATL	Disseminated	1-mo history of weakness, fatigue, and weight loss. 5 d: shortness of breath, bilateral pleural effusion, ascites, and severe abdominal pain Died	CSF AgCL: 1:640 *C neoformans* isolated from blood, BALF, and ascitic fluid	AmB deoxycholate + 5FC
Desai et al [35]	1	NA	NA	ATL, *Pneumocystis* pneumonia	Pulmonary	NA	NA	FCZ
Kawamoto et al [36]	1	F/85	NA	NA	Lymphadenitis	Lymphadenitis	NA	NA
Debourgogne et al [25]	2	NA	French Guiana	1 with histoplasmosis	NA	NA	NA	NA
Kohno et al [17]	6	5 F/37– 77	Japan	1 diabetes 1 bladder tumor 1 CKD	Pulmonary	1 cough 1 chest pain 1 fever and lymphadenopathy 3 asymptomatic	Chest X-ray: 2 multiple nodules 4 pulmonary infiltrates	NA
Sasayama et al [19]	1	M/75	Japan	Chronic bronchitis, bladder tumor	Disseminated	Fever, cough, inguinal lymphadenopathy Died	Serum AgCL:1:20 Diffuse pulmonary infiltrates, anemia, thrombocytopenia, elevated transaminases	Miconazole, FCZ, AmB + 5FC
Hirose et al [13]	1	M/70	Japan	None	Meningeal	Fever, headache, meningeal signs Cured	CSF with pleocytosis, low glucose, high proteins; *C neoformans* isolated from CSF	NA
Kinjo et al [16]	1	64	Japan	Hemodialysis	Pleural	Cough and pleural effusion	*C neoformans* isolated from pleural effusion	NA
Rhew et al [37]	1	M/83	Iowa, USA	Chronic ATL, pneumonia by *Pneumocystis jirovecii*, *Mycoplasma pneumoniae*, and *Mycobacterium avium* complex	Pulmonary	Cough without fever, pleural effusion, anemia, and leukopenia Obstructive lesion in the trachea Died	*C neoformans* isolated from the trachea	AmB, FCZ
Taguchi et al [21]	1	M/70	Japan	Waldenstrom macroglobulinemia	Pulmonary	Cervical nodes, bilateral pleural effusion Cured	Serum AgCL 1:16 *C neoformans* (serotype A) isolated from pleural effusion	Miconazole (intrapleural and IV), 5FC
Funakawa et al [12]	1	M/73	Taiwan, China	NA	Meningeal	NA	NA	FCZ + 5FC
Tashiro et al [22]	2	M/66	Oita, Japan	Total gastrectomy, ATL smoldering *Pneumocystis jirovecii*	Meningeal	Fever, headache, pulmonary infiltrates Died	*C neoformans* isolated from CSF	AmB + 5FC, FCZ, cotrimoxazole, pentamidine
		M/55	Oita, Japan	ATL	Pulmonary	Cough, lung cavitation. Cure	*C neoformans* isolated from the lung	FCZ, lobectomy
Chalkias et al [26]	1	M/82	Caribbean	T-cell lymphoma	Disseminated (meningeal, pulmonary)	Fever, hemoptysis, right pulmonary consolidation with nodular lesions; aphasia and confusion Died	*C neoformans* isolated from CSF and from BALF; CSF and serum AgCL >1:64	Piperacillin-tazobactam, AmB deoxycholate + 5FC
Clark et al [11]	1	NA	Okinawa, Japan	ATL	Meningeal	NA	NA	NA
Beenhouwer et al [34]	1	M/38	New York, USA	Alcoholism, hepatitis C, and liver cirrhosis, carrying a portocaval shunt	Recurrent meningeal	Fever, photophobia, neck stiffness Cured	*C neoformans* isolated from CSF	Ceftriaxone, AmB 660 mg in 14 d, then FCZ 400 mg/d for 2 mo
Adedayo et al [33]	1	M/31	Western India	None	Pulmonary	Skin lesions, cough, and hemoptysis and crackles at the base of the right lung Cured	*C neoformans* identified from the lung	AmB 26 mg/d for 3 wk, then FCZ 400 mg/d for 3 wk
Inoue et al [14]	1	M/46	Kyushu, Japan	Acute ATL, thymoma	Pulmonary	Pulmonary nodules Cured	Histopathology of lung showed yeasts of *Cryptococcus*	Surgery and FCZ 100 mg/d for 2 mo
Kaneko et al [15]	1	M/73	Japan	Chronic ATL	Pulmonary	Altered mental status and fever Died	*Cryptococcus* identified from lung tissue	Piperacillin 6 g, panipenem/betamipron 2 g and FCZ 200 mg for 12 d
Miyoshi et al [18]	1	M/66	Kochi, Japan	Chronic ATL	Skin and lymph nodes	Fever, inguinal lymph nodes, erythematous rash on the trunk and extremities Cured	*Cryptococcus* spp identified from the skin and lymph nodes.	FCZ + AmB for 23 d
Suzuki et al [20]	1	F/80	Japan	Adult T-cell leukemia	Pulmonary and hepatic	Jaundice, abnormal liver function tests, and leukocytosis; lung consolidation and cavitation Died	*Cryptococcus* spp isolated from sputum cultures Multiple granulomas were observed from the autopsy of the liver, consistent with cryptococcal bodies	NA

Abbreviations: 5FC, flucytosine; ADA, adenosine deaminase; AgCL, cryptococcal antigen testing; AmB, amphotericin B; ATL, T-cell leukemia/lymphoma in adults; BALF, bronchoalveolar lavage fluid; CFU, colony-forming units; CKD, chronic kidney disease; CSF, cerebrospinal fluid; F, female; FCZ, fluconazole; GCS, Glasgow Coma Scale; HBP, high blood pressure; IV, intravenous; M, male; NA, not available; OP, opening pressure, TFG, time of fungal growth; USA, United States; WBC, white blood cells.

Five of 7 Peruvian patients came from Andean and jungle regions. Mean age was 59 ± 8.9 years; 5 subjects were male and most of them had 1 comorbidity. *Cryptococcus neoformans* was the etiologic agent isolated in all subjects.

Meningeal cryptococcosis was detected in 6 of the 7 patients (85.7%). In the remaining patient, a lumbar puncture study was not performed, preventing the identification of meningeal cryptococcosis. Among the 6 patients in whom we identified meningeal cryptococcosis, only 1 patient had disseminated disease with cryptococcemia, pulmonary, gastrointestinal, and meningeal involvement. A baseline cerebrospinal fluid (CSF) latex agglutination antigen titer >1:1024 was found in 50% (n = 3/6) of the subjects, who also had altered mental status. CSF features of the 6 patients disclosed elevated opening pressure in 83.3% (n = 5), with a mean value of 33 cm H_2_O; 50% (n = 3) had India ink positive test; mean glucose level was 13 mg/dL (range, 2–29 mg/dL); median protein level was 100 g/L (range, 14–300 g/L), and median CSF white blood cell count was 148 cells/μL (range, 11–410 cells/μL). CSF fungal burden was measured in 50% (n = 3/6) of the subjects; the mean value was 7.39 ± 0.1 colony-forming units log_10_/mL and the mean isolation time was 5 ± 2.3 days. Lower fungal burden was observed in the remaining 50% (n = 3/6) of the subjects, with 7–10 days of growing time.

All 6 subjects received amphotericin B deoxycholate (0.6–1 mg/lg/day) plus fluconazole (800 mg/d) until obtaining a negative CSF culture. In 2 subjects, high opening pressure was maintained, and the CSF cultures remained positive after 21–40 days of treatment; both subjects died with *Acinetobacter* spp bacteremia.

Twenty-one publications with information on 29 patients with HTLV-1 and cryptococcosis disease were available for review. Most publications (12 [41.38%]) included Asian people. Data description was insufficient in 5 publications. The treatment was not standard in all cases. Amphotericin (AmB), fluconazole, 5-flucytosine (5-FC), miconazole, AmB plus 5-FC, fluconazole plus 5-FC, and lung surgery were prescribed. Survival information was available for 16 patients, with 7 of them remaining alive.

## DISCUSSION

We report here 7 HIV-uninfected patients coinfected with HTLV-1 and cryptococcosis. In contrast to what has been reported from Asia and the Caribbean on this coinfection, where patients had essentially pulmonary involvement, our patients presented with meningeal involvement. However, more disseminated disease was not regularly investigated in all these studies including ours. Interestingly, patients coinfected with HTLV-1 and cryptococcosis received different treatment regimens.

Peru is an endemic country for HTLV-1, which has been reported in 2.5%–10% of Quechua ethnic groups from the mountains of Peru [38, 39] and in 1.9%–5.9% of groups of native communities from the jungle region [40, 41]. Most of the 7 cases of this report were from the Andean and jungle regions of Peru. The precise etiology behind the elevated prevalence of HTLV-1 infection among Andean populations remains uncertain; certain researchers have hypothesized that factors such as geography, culture, or human leukocyte antigen types may contribute to this phenomenon [42].

HTLV-1 infection may increase susceptibility to opportunistic infections due to impaired immune response caused by regulatory protein expression. P30, a regulatory protein, reduces transcription in Toll-like receptor 4, leading to the inhibition of proinflammatory cytokines like monocyte chemoattractant protein 1, tumor necrosis factor α, and interleukin 8 [43]. This suggests a potential mechanism for the heightened vulnerability to various pathogens in individuals with HTLV-1 infection.

The report highlights meningeal involvement in HTLV-1 and cryptococcosis cases, with specific characteristics such as pleocytosis, low glucose, and high protein concentration. India ink test results were negative in 50% of cases, and the fungal burden was notably high. In HIV-associated cryptococcal meningitis, the CSF white cell count is usually low and may even be normal. In contrast, like in other case series in patients with HTLV-1, we found pleocytosis in the CSF [13, 22, 26, 34]. This finding needs to be corroborated in larger studies. Disseminated cryptococcosis was not commonly investigated in this report or in others. CSF fungal burden data have not been extensively studied. In PWH, a fungal burden >4.5 colony-forming units log_10_/mL is linked to early clearance failure, but its correlation with antigen titers is unclear [44, 45]. Further research is needed to understand these associations fully.

Mortality is higher in HIV-uninfected and nontransplant patients compared to PWH and transplant recipients [46, 47]. In our description, 3 of 7 patients died. The mortality of previous reports was 56%.

In conclusion, we report 7 patients with HTLV-1 and cryptococcal infection. We recommend that patients with cryptococcosis should be tested for HTLV-1 infection in endemic areas for this human retrovirus.

## Notes

***Acknowledgments*.** We thank the staff of the Clinical Mycology Laboratory of Tropical Institute Alexander von Humboldt for their help in the sample processing.

***Author contributions.*** Conceptualization and methodology: F. C.-V. and B. B. Software: F. C.-V. Clinical data: B. B., E. G., and C. S. Writing—original draft preparation: F. C.-V. and B. B. Writing—review and editing: C. S., B. B. and E. G. Visualization: F. C.-V. and C. S. Comments on the manuscript: All authors.

***Ethics statement.*** This work was approved by the Institutional Research Ethics Committee of the Continental University (approval document number 0149-2023-CIEI-UC). The current investigation involved a secondary data analysis, and as such, there was no direct interaction with the participants. The data were obtained from medical and laboratory records during the standard operations of the Alexander von Humboldt Tropical Medicine Institute. The collection and analysis process of the information employed internal codes, whereby all identification details were stored separately and did not conform part of the analysis.
